# Rafting behaviour of seabirds as a proxy to describe surface ocean currents in the Balearic Sea

**DOI:** 10.1038/s41598-018-36819-w

**Published:** 2019-01-10

**Authors:** A. Sánchez-Román, L. Gómez-Navarro, R. Fablet, D. Oro, E. Mason, J. M. Arcos, S. Ruiz, A. Pascual

**Affiliations:** 1 Instituto Mediterráneo de Estudios Avanzados, IMEDEA (CSIC-UIB), C/ Miquel Marquès, 21, Esporles, 07190 Illes Balears Spain; 2 University Grenoble Alpes, CNRS, IRD, IGE, Grenoble, 38400 France; 3 labSTICC, TOMS, Brest, 29238 France; 4 Applied Physics Laboratory, University of Washington, Seattle, Washington USA; 5 SEO/BirdLife, Marine Programme, Barcelona, Spain

**Keywords:** Marine biology, Physical oceanography

## Abstract

Spatio-temporal variability of surface geostrophic mesoscale currents in the Balearic Sea (western Mediterranean) is characterized from satellite altimetry in combination with *in-situ* velocity measurements collected, among others, by drifting buoys, gliders and high-frequency radar. Here, we explore the use of tracking data from living organisms in the Balearic Sea as an alternative way to acquire *in-situ* velocity measurements. Specifically, we use GPS-tracks of resting Scopoli’s shearwaters *Calonectris diomedea*, that act as passive drifters, and compare them with satellite-derived velocity patterns. Results suggest that animal-borne GPS data can be used to identify rafting behaviour outside of the breeding colonies and, furthermore, as a proxy to describe local sea surface currents. Four rafting patterns were identified according to the prevailing driving forces responsible for the observed trajectories. We find that 76% of the bird trajectories are associated with the combined effects of slippage and Ekman drift and/or surface drag; 59% are directly driven by the sea surface currents. Shearwaters are therefore likely to be passively transported by these driving forces while resting. The tracks are generally consistent with the mesoscale features observed in satellite data and identified with eddy-tracking software.

## Introduction

The relatively recent development of miniaturized bio-logging devices has allowed the study of fine-scale distribution and behaviour of seabirds while they are travelling, feeding or searching for food at sea^1^. Aside from the ecological and conservation implications, such seabirds’ trajectories can be used as proxies to investigate the dynamics of marine systems and their spatiotemporal evolution. For instance, previous studies^2^ assessed wind patterns and atmospheric conditions in the lower atmosphere by analysing foraging trips of the great frigatebird *Fregata minor* whilst^3^ identified oceanic fronts in the uppermost part of the ocean water column according to the locations of feeding and foraging by Cory’s shearwaters *Calonectris borealis*.

However, most of the studies in the literature are focused on: (i) analysis of long range flights, where birds are travelling at high speeds over long periods; (ii) the identification of foraging hotspots or; (iii) the development of habitat models. Conversely, the rafting behaviour of seabirds has been poorly investigated. Recently^4^, used the global positioning system (GPS) to track breeding Northern Gannets *Morus bassanus* in order to study the incidence of rafting behaviour on foraging trips; rafting was observed in 62% of the breeding foraging trips that were monitored. During rafting, when birds are settled on the sea surface, they tend to be passive drifters that are carried by ocean surface currents, namely geostrophic and ageostrophic flows. The latter are driven by direct stress imparted by the local wind (i.e. Ekman and/or Stokes drifts), as shown by^5^. These authors used streaked shearwaters *Calonectris leucomelas* as Lagrangian sensors that emulated drifting buoys to investigate the Oyashio ─ Tsugaru Warm Current confluence near Japan. They compared currents deduced from seabird drift movements with ocean surface currents derived from *in-situ* observations and satellite data and were able to demonstrate that animal-borne GPS data can be a cost-efficient tool to observe ocean surface currents. Later^6^, assimilated these seabird data and ship drift data into an operational ocean model forecast system, from which they were able to demonstrate refinement in the simulated gyre mode events of the Tsugaru Warm Current.

The Mediterranean Sea is a semi-enclosed basin where many phenomena such as deep convection, thermohaline circulation, water mass interaction and mesoscale dynamics can be observed and studied at scales smaller than in the large scale basins^7^. Thus, physical mechanisms and their interactions with biological processes can be more easily monitored and understood. Sea surface variability in the Mediterranean Sea can be realistically monitored and characterized from satellite altimetry^8–12^. This approach provides surface geostrophic currents at relatively large spatial scales. Satellite-derived geostrophic currents are normally validated through comparison with *in situ* velocity measurements collected, among others, by drifting buoys^13–16^, gliders^17–20^, vessel-mounted Acoustic Doppler Current Profilers^10,20^ and High-Frequency radars^21,22^ in the uppermost part of the water column. These comparisons provide an improved representation of the surface circulation.

Drifter measurements are a particularly valuable tool for validating altimetric-derived velocities in the open sea^23^. In this study, we compare the satellite-derived velocity patterns in the Balearic Sea (western Mediterranean Sea) with the paths followed by Scopoli’s shearwaters when they are at rest on the sea surface and hence act as passive drifters (emulating drogue-less drifting buoys) in an attempt to determine whether animal-borne GPS data can be used as a proxy to describe sea surface currents in the area. Such drifters, without a holey-sock drogue (usually placed at around 15 m depth) are strongly affected by the direct local wind and, to a lesser extent, by surface waves acting on the upper part of the drifter that protrudes above the sea surface^16^. As a result, drifters are not perfect Lagrangian instruments and may not accurately follow the near-surface currents^24^. The relative movement of drifters with respect to currents under the direct action of wind and waves is referred to as “slippage”. Drifters are also affected by the wind-driven Ekman currents, and the same applies to seabirds while resting. Thus, we use the outputs of the Cross-Calibrated Multi Platform version 2 (CCMPv2) gridded surface L3 ocean vector wind analysis product^25^ to investigate both the wind-induced slip and wind-driven Ekman drift of the tracked seabirds.

**Figure 1:**
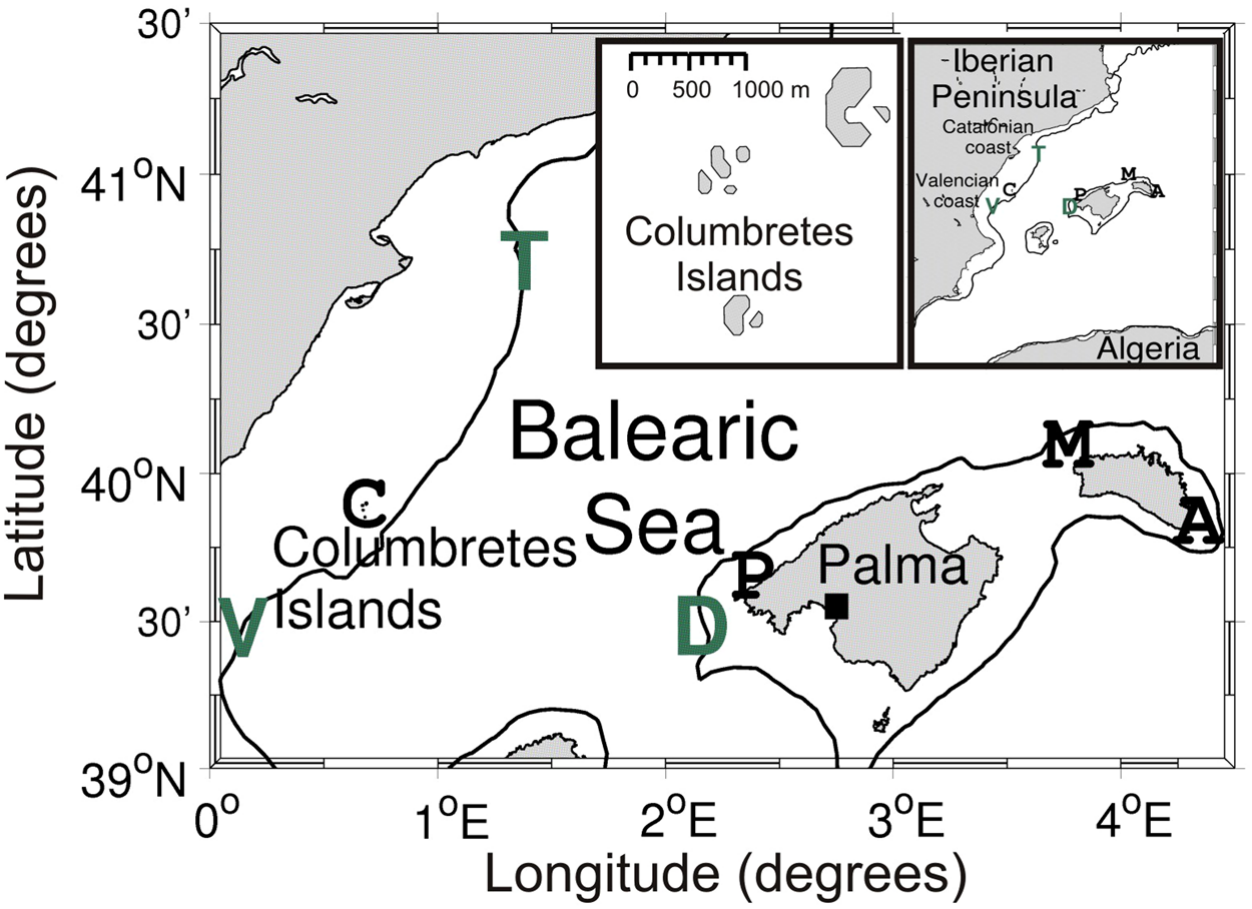
Map of the Balearic Sea region showing the location of the breeding colonies of (A) Illa de l’Aire and (M) Cala Morell (Menorca Island); (P) Illot de Pantaleu (Mallorca Island) and (C) Columbretes (Columbretes Islands). Also, the surface buoys (in green) of (T) Tarragona, (V) Valencia and (D) Dragonera are displayed. These buoys are located over the 200 m isobath (black line) delimiting the continental/insular shelf-slope. The inset map at the top right corner displays the full study region whilst the inset map on the left shows a zoom of the Columbretes archipelago.

**Figure 2:**
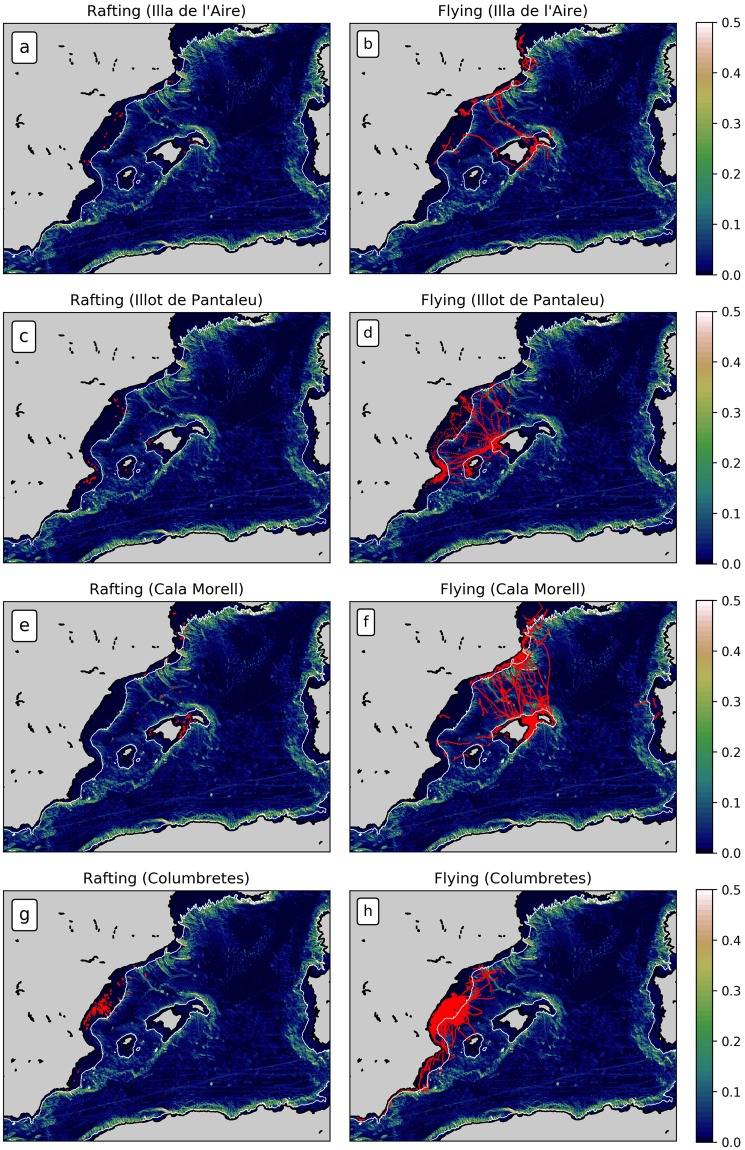
Rafting behaviour (red patches, left column) and foraging trips (red lines, right column) of individual seabirds tracked during each campaign according to the speed criterion described by^26^. White lines show the 200-m isobath that delimits the continental/insular shelf-slope; topographic data from the Shuttle Radar Topography Mission [SRTM; e.g., *Smith and Sandwell*, 1997]. Colors indicate the slope (m) of the SRTM bathymetry in the western Mediterranean.

The aim of this study is to investigate the feasibility of using the rafting behaviour of seabirds as a proxy to describe surface currents in the Balearic Sea (Fig. 1). During their foraging trips, these birds habitually rest on the sea surface (Fig. 2), where they tend to be passive drifters. The final goal of this work is to contribute to the understanding of marine system’s dynamics and their spatio-temporal evolution.

## Results

### Validation of wind patterns and geostrophic velocities

**Figure 3:**
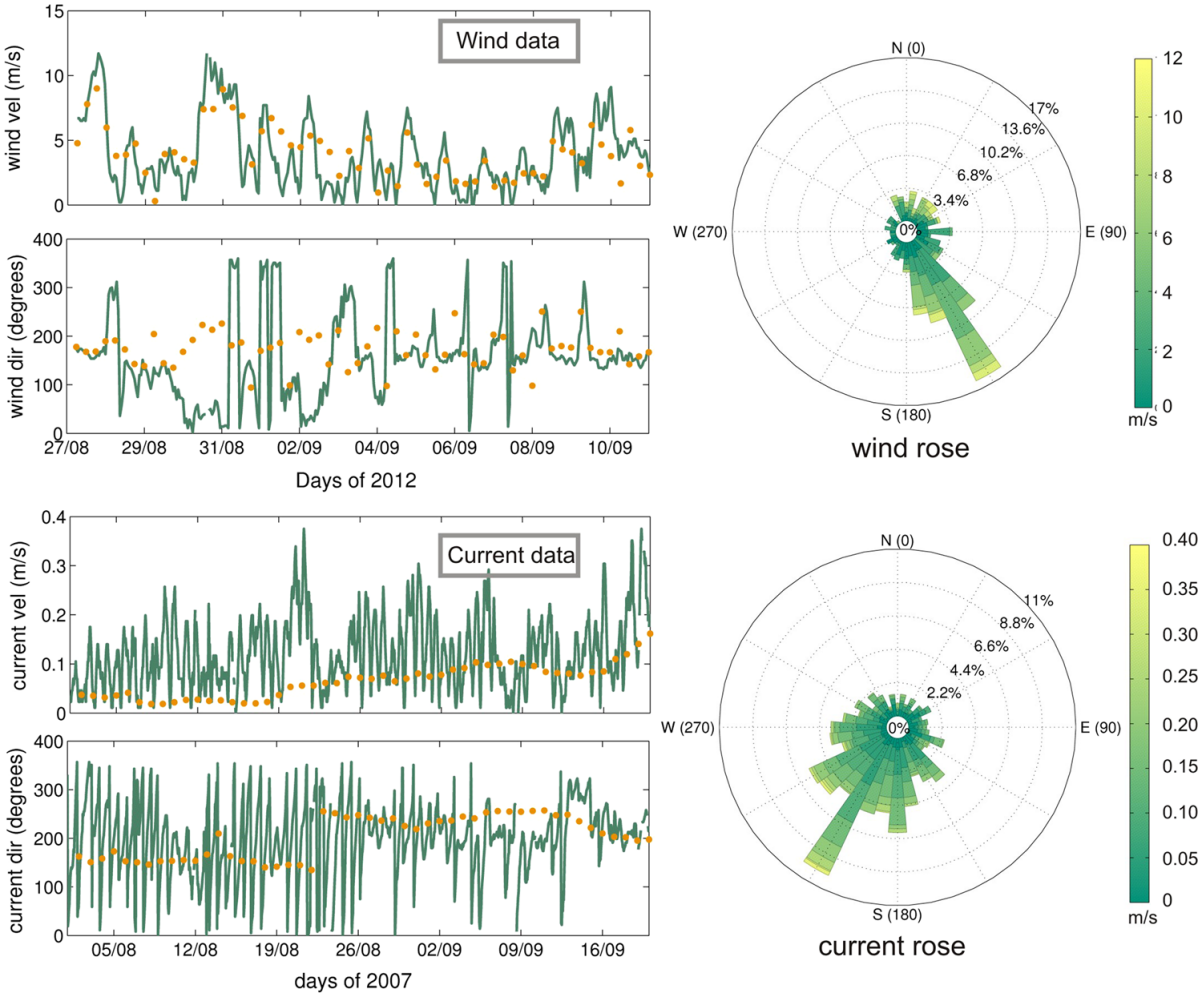
Comparison of CCMP winds and satellite-derived altimetry data with *in situ* observations: validation of wind speed (m s^−1^) and direction (degrees) from CCMP (orange dots in the two upper panels, respectively) obtained for the 2012Colu campaign (Table 1) by using *in situ* measurements (green lines) collected from the surface Valencia buoy (39.52°N/0.21°E). Geostrophic velocities (current speed, m s^−1^, and direction, degrees) derived from satellite altimetry (orange dots in the two lower panels, respectively) obtained for the three campaigns conducted in 2007 (Table 1) are also validated by using *in situ* measurements (green lines) collected from the surface Tarragona buoy (40.68°N/1.47°E). The wind and current rose diagrams (m s^−1^) are derived from the two aforementioned buoy datasets, respectively. Notice that *in situ* wind direction indicates the origin of the local wind whilst surface current direction denotes where it is going.

Prior to comparison of seabird drift trajectories with local wind patterns and sea surface geostrophic currents, we validated the wind product and altimetry data in order to obtain reliable spatial patterns. We used *in situ* current and wind data obtained from three surface buoys deployed along the eastern Spanish mainland coast and the Balearic archipelago (Fig. 1). We interpolated the wind and current fields to the position and time of the *in situ* measurements and computed root mean square errors (RMSE) for both the altimetric and wind product data with respect to the *in situ* observations. Figure 3 shows an example of the comparison between: (i) the wind product outputs (wind speed and direction) obtained for the campaign of 2012 Colu (see Table 1), and the wind data collected at 3 m above the sea surface by the Valencia buoy at 39.52°N/0.21°E (upper two panels and upper rose plot); and (ii) surface geostrophic currents (current speed and direction) from the three campaigns of 2007, and total currents measured at 3 m depth by the Tarragona buoy at 40.68°N/1.47°E (lower two panels and lower rose plot in Fig. 3). Note that *in situ* wind direction indicates the origin of the local wind whilst surface current direction denotes its direction of flow.

**Table 1:** Incidence of rafting. Number of total tracked birds, tracked birds providing data, location of the breeding colonies and time period analysed for the six campaigns included in this study.

CAMPAIGN	Breeding colony	Lat (°N)	Lon (°E)	Tracked birds	Tracked birds providing data	No. GPS fixes (rafting)	Period analyzed
2007Aire	Illa de l’Aire (Menorca Island)	39.80	4.24	6	4	1053	06/08/07–12/08/07
2007Pant	Illot de Pantaleu (Mallorca Island)	39.57	2.34	10	6	837	19/08/07–29/08/07
2007More	Cala Morell (Menorca Island)	40.05	3.88	14	9	853	04–09/08/07 10–18/09/07
2010More				30	25	2238	18/06/10–12/07/10
2011Colu	Columbretes (Columbretes Islands)	39.89	0.68	22	13	188	14/07/11–26/07/11
2012Colu				24	18	6924	23/08/12–11/09/12

The number of rafting GPS fixes used in each campaign is also included.

*In situ* wind velocities collected by the Valencia buoy (uppermost panel in Fig. 3) exhibit daily fluctuations, with maximum values larger than 10 m s^−1^ towards the end of August 2012 which are significantly lower during the first days of September. Wind speed extracted from the wind product (orange dots) shows a similar temporal pattern with systematically lower (larger) velocities during the periods of high (low) *in situ* wind speed. As a consequence, the wind product has a standard deviation of 1.94 m s^−1^, 23% lower than that obtained for the *in situ* measurements (2.52 m s^−1^). This is due to the buoy recording higher frequency variability than that retrieved from the wind product with its longer time resolution (hourly vs. 6-hourly interval measurements, respectively). Moreover, the spatial resolution of the wind product (¼° × ¼°) means that the wind spatial variability is also degraded, translating into larger discrepancies with the *in-situ* buoy measurements. These factors also apply to the wind direction comparison. *In situ* winds mainly blew from the south south-east (wind origin ranging between 140 and 175 degrees, see second panel at top and wind rose in the upper right side of Fig. 3). Again, the wind product resembles the *in situ* wind measurements with origins ranging mainly between 150 and 190 degrees. As a result, we obtain an RMSE between both datasets of 1.76 m s^−1^ for the wind speed and 44 degrees for the wind direction. The correlation coefficients (p value < 0.05) are, respectively, 0.71 and 0.49, highlighting the quality of the wind product data used to compare with the seabird tracks.

Total *in situ* current velocities collected by the Tarragona buoy between August and September 2007 (third panel from top in Fig. 3) have a more chaotic pattern with fluctuations ranging between 0.2 and 0.3 m s^−1^. This is due to the high-frequency variability of the surface currents which is captured by the buoy (hourly interval measurements). By contrast, these fluctuations are not observed in the geostrophic currents derived from altimetry since they are obtained from daily data. In general, satellite-derived currents have lower values than the *in situ* total velocities; this is related to the presence of ageostrophic components in the latter which are not measured by altimetry. However, both datasets appear to have the same temporal behaviour at monthly scale with an RMSE of 0.07 m s^−1^. The *in situ* total current direction (lowermost panel and current rose in the lower right side of Fig. 3) also has large temporal variability. It ranges roughly between 150 and 250 degrees so that surface currents flow mainly towards the south south-west. The surface geostrophic currents derived from altimetry resemble this general pattern, although they have much lower temporal variability with a mean direction of nearly 170 degrees (towards the south) until 20 August, and then shifts towards the southwest (current direction between 200 and 250 degrees) until the end of the analysis period. These discrepancies give an RMSE of 48 degrees between the two datasets.

### Identifying the driving forces of seabird drift

**Figure 4:**
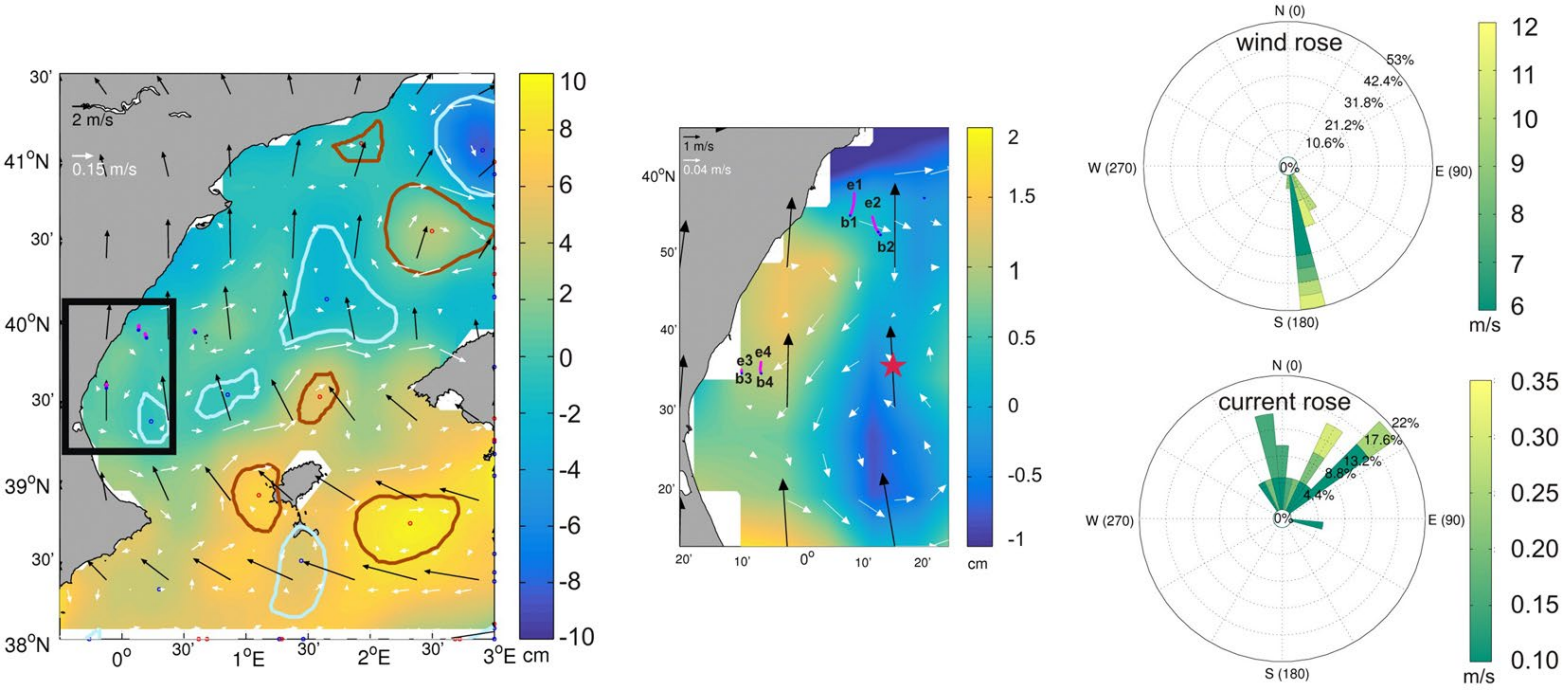
An example map showing a 6-hours snapshot (left panel) on 27 August 2012 of ADT (cm) from altimetry and the corresponding 6-hour mean wind field as obtained from CCMP between 03:00–09:00 hours, together with several seabird tracks driven by the local wind. White arrows denote geostrophic currents (m s^−1^) from altimetry; black arrows are associated with the wind data (m s^−1^). Pink lines represent the four seabird tracks observed over a period of 6 hours, according to the wind product data time used to construct the composite. Blue dots mark the beginning of the tracks. The birds are from the Columbretes colony (see Fig. 1 and Table 1). Brown and pale-blue regions correspond respectively to the external limits of the anticyclonic and cyclonic eddies as identified by the eddy tracker. The middle panel shows a zoom of the area inside the black square displayed in the left panel that shows the four bird trajectories. The ends of the trajectories are indicated by ex whilst bx indicates their beginning; *x* denotes the number of the track (1 to 4). Finally, rose diagrams on the right side of the figure (m s^−1^) indicate the speed and direction of *in situ* local winds (upper) and local currents (lower) as measured by the surface Valencia buoy (red star in middle panel) at 39.52°N/0.21°E that is used to validate both the wind product and altimetry data.

**Figure 5:**
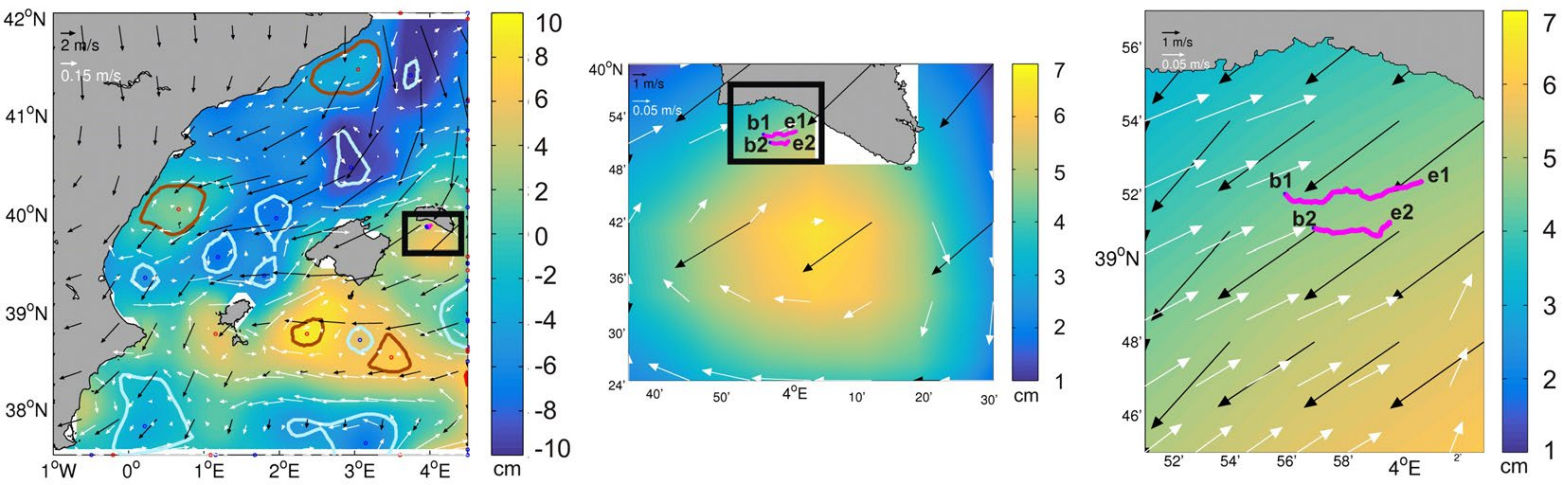
Same as Fig. 4 but for a different 6-hours snapshot on 7 August 2007 showing two parallel tracks that follow an anticyclonic eddy south of Menorca island. In this case, the birds are from the Illa de l’Aire breeding colony (Menorca).

The rafting behaviour of every tracked seabird was visually compared with the local wind and surface geostrophic velocity patterns in order to establish the driving force of the observed seabird drift movement in the Balearic Sea. As altimetry maps and wind data have different time scales (resp., daily vs. 6-hourly), we produced 6-hourly composites according to the wind data time resolution by using the same altimetry map for the four composites produced for a given day. We then added the seabird trajectories found within a time window of 6 hours centred around the wind data time used to construct each composite. Examples of these composites are provided in Figs 4 and 5. We distinguish between four different rafting patterns according to the prevailing driving force of the seabird tracks: (i) trajectories only driven by the local surface wind (namely, slippage and Ekman drift which should be 45 degrees to the right of the wind direction) where no evidence of current drag is observed; (ii) trajectories only driven by the surface ocean currents with negligible wind effect; (iii) trajectories driven by both local winds and surface currents (mixture of slippage, Ekman drift and current drag); and (iv) trajectories driven by other factors not addressed in this study, such as birds resting on ships or flying with velocities under the 0.5 m s^−1^ “apparent” speed threshold (see below). In this case, the observed tracks cannot be associated with either wind effects nor surface current drag. Table 2 summarizes the results obtained for the 405 trajectories from the 75 tracked individuals, and which provided the seabird tracks that were estimated according to the criteria described by^26^. 85% of the observed drift trajectories (344 from a total of 405) lasted between 3 and up to 6 hours (3 hours is the minimum time threshold considered in order to ensure a valid comparison with both sea surface velocity patterns derived from altimetry and wind fields from the CCMP wind product; see Methods section). The remaining 15% of trajectories (61 from a total of 405) extended between 7–10 hours and were associated with individuals from the colonies located in the islands of Mallorca and Menorca (see Table 1 and Fig. 1).

**Table 2:** Tracked individuals and rafting trajectories identified from each campaign according to the driving force (percentage of total trajectories analysed in the campaign).

Campaign	Trajectories	Trajectories Driven By			
		Surface Wind	Surface Currents	Surface Wind + Currents	Others
2007 Aire	35 (4 individuals)	8 (23%)	8 (23%)	12 (34%)	7 (20%)
2007 Pant	43 (6 individuals)	12 (28%)	10 (23%)	10 (23%)	11 (26%)
2007More	37 (9 individuals)	13 (35%)	6 (16%)	7 (19%)	11 (30%)
2010 More	107 (25 individuals)	40 (37%)	17 (16%)	27 (25%)	23 (22%)
2011 Colu	17 (13 individuals)	4 (24%)	4 (24%)	7 (41%)	2 (11%)
2012 Colu	166 (18 individuals)	49 (29%)	26 (16%)	46 (28%)	45 (27%)
TOTAL	405 (75 individuals)	126 (31%)	71 (18%)	109 (27%)	99 (24%)

We identified 126 trajectories (from a total of 405) that followed paths according to the local wind patterns, meaning that seabirds at rest on the sea surface were likely to be driven by both the direct slip and Ekman drift rather than the surface currents. These represent nearly 31% of the total number of trajectories. This percentage oscillates between 23% and 37% for the different campaigns. Figure 4 displays a 6-hourly snapshot with four tracks corresponding to four different individuals. All follow a roughly northward path close to the eastern Spanish mainland coast. Blue dots (bx) indicate the starts of the tracks. In this snapshot, the local wind was blowing from the south (black arrows in Fig. 4). This fact is corroborated by the wind data collected by the Valencia buoy (red star in the zoom subplot representing the area inside the black square) and is reflected in the *in situ* wind rose on the right side of the figure. These data show local winds blowing mainly from 175 degrees. The strong visual agreement between seabird drift and the wind field indicates that the movement of the four individuals was driven by slippage and Ekman drift, both derived from the action of local winds rather than local surface currents, which would actually would have pushed the birds south-east and south-west (according to their location) driven by the anticyclonic eddy centred at 39.75°N as derived from the geostrophic currents for that day.

The trajectories driven by the surface currents only, i.e., those reflecting the mesoscale features observed in altimetry in the Balearic Sea, represent around 18% (71 tracks) of the total. This percentage varies from 16% to 24% depending on the location of the seabirds and the presence of eddies or other fine-scale structures (order 30–100 km) in the area. One example of these current-driven tracks is displayed in Fig. 5, which shows a 6-hours snapshot with two seabird tracks running parallel to each other south of Menorca. These tracks are associated with two individuals that are moving simultaneously, close to the edge of an anticyclonic eddy (clockwise rotating) centred at 4° E and reflecting the geostrophic currents generated by the eddy close to the coast (white arrows in Fig. 5). The local wind here is blowing from the north-east such that Ekman drift should roughly propel the birds towards the west south-west; i.e. in the opposite direction to the actual trajectories. In this case, no validation with *in situ* measurements was made because of the lack of surface buoys in the area.

In addition, we visually identified 109 trajectories driven by both direct slip and Ekman drift, and surface current drag. These represent 27% of the number of trajectories analysed. Considering the different campaigns, this percentage oscillates between 19% and 41%. Finally, the remaining 99 trajectories could not be associated with either the local wind field or surface current pattern. These represent around 24% of the trajectories investigated, varying between 11% and 30% depending on the campaign and the area where birds rest on the sea surface. They could be related to the alternation between flights and stops.

We also used an automated eddy tracker to assess the relative positions of eddies in the Balearic Sea and seabird tracks in an attempt to establish a relationship between drifting behaviour and the presence of cyclonic and anticyclonic features. We differentiate between tracks located either at the periphery or inside eddies, and tracks passing between eddy pairs whose opposing rotational motions lead to strong frontal shear. In both cases, birds can be passively transported by the geostrophic currents. We found that only 6% of the trajectories were located in frontal regions between eddy pairs whilst around 17% of the tracks were observed inside the eddy interiors as identified by the eddy tracker. An example of this eddy identification is provided in Figs 4 and 5. In contrast, almost 77% of the tracks were located in regions away from the dynamical influence of observed eddies and so no statistically significant correlations were obtained. This outcome can be partially explained by the fact that satellite altimetry only captures the geostrophic component of the surface currents, whilst birds resting on the sea surface are also driven by slippage and Ekman drift that has been proven to have an important impact on the observed tracks in the area of study (31% of the analysed trajectories were driven only by the local wind), and Stokes drift derived from the ocean surface waves. The latter quantity has not been investigated here.

## Discussion

The foraging trips and feeding areas of the Scopoli’s shearwaters along the eastern Iberian coasts and the Balearic Islands are likely influenced by the location of their breeding colonies, as reflected in the flying trajectories (Fig. 2, right column)^27–29^, and also by the intensity of local winds: favourable wind windows allow longer trips with lower energetic costs^30^. This fact in turn determines the areas where rafting behaviour is observed. 100% of the individuals provided rafting tracks according to the criteria used here (see the Methods section). Seabirds from the breeding colonies settling near Menorca (Illa de l’Aire and Cala Morell, see Table 1) mainly searched for food along the insular coast. The same occurred off in the northern coast of Mallorca. Other seabirds travel across the Balearic Sea towards the north-easternmost coasts of the Spanish mainland (Catalonian region) and the southeastern coasts of France (Fig. 2, panel b & f). On the other hand, birds from the Illot of Pantaleu (southwest of Mallorca) mostly travelled between their breeding colony and the eastern coasts of the Iberian Peninsula south of the Catalonian region (see panel d in Fig. 2). Finally, individuals from Columbretes foraged and fed near the Spanish mainland coasts near the Valencian region (Fig. 2, panel h), and only a reduced number of the birds reached the Balearic Islands and the Catalonian regions. As a consequence, different *rafting regions* can be identified according to the location of the colonies: (i) the eastern Iberian coasts, (ii) the southwest and northeastern coasts of Mallorca, (iii) the southwest and northwest coasts of Menorca and, to a certain extent, (iv) the southernmost coasts of France (see left column in Fig. 2). These regions agree with the predicted areas for foraging and feeding by Scopoli’s Shearwater with an integrated habitat model in the Balearic Sea reported by^26^. These authors stated that the foraging areas of the shearwaters respond to complex bio-physical coupling, such as the presence of frontal features with large ocean productivity. They found three important foraging hotspots located along the continental shelf-slope of the Iberian Peninsula: the Gulf of Lions, the waters off the Catalonian region and the coasts of the Valencian region. All three have been identified here as foraging regions (see Fig. 2). Also, most of the observed trajectories associated with rafting behaviour occurred where feeding was possible due to the presence of relatively shallow and nutrient-rich waters (note that the bottom slope in these regions is practically null) fed by the interaction between the Northern Current coming from the Gulf of Lions^31^; and river discharges (mainland and insular, e.g. the Ebro river). Thus the rafting behaviour of Scopoli’s shearwaters in the study area probably takes place after or between feeding episodes. Overall, rafting periods represent around 10% of the total seabird activity investigated here (according to the criteria used to identify the rafting behaviour described below). Individuals therefore spent most of their time (90%) travelling between the colonies and feeding areas (foraging trips), feeding or in the colony for incubation and chick-rearing (out of our scope). Namely, we found that Scopoli’s shearwaters tend to spend up to 6 hours resting at the sea surface, as reflected in 85% of tracks analysed. This involving 344 trajectories from a total of 405. Occasionally, these time periods extend above 6 hours and up to 8–10 hours (15% of total trajectories investigated).

This preliminary analysis suggests that Scopoli’s shearwater drift movements in the Balearic Sea are principally induced by ocean surface currents and local winds. Thus, shearwaters are likely to be passively transported by these driving forces while resting. Overall, 76% of the seabird drift trajectories analysed here (306 of a total of 405) were visually associated with slippage and Ekman drift and/or surface drag. Ekman drift should theoretically transport the seabirds at a 45 degrees angle to the right of the wind in the northern hemisphere. However, we visually identified Ekman currents with angles less than 30 degrees; this is in accordance with the angles reported by^16,24^ for drogue-less drifters in the Mediterranean Sea.

The prevalence of one driving force over another depends on the meteorological conditions (wind intensity) and/or the presence of mesoscale features such as eddies or surface currents in the area. Furthermore, we found that the seabird tracks driven by the surface currents, or the combination of the former with local winds, reconstructed some parts of the mesoscale features observed from satellite derived data and identified with the eddy-tracker tool^32^, thus indicating that seabird drift data in the Balearic Sea could be used to monitor regional currents^5^. The latter authors reported that seabird data from streaked shearwaters were used to reconstruct a characteristic anticyclonic gyre mode during summer in the eastern region of the Tsugaru Strait; the tracked individuals in the study spent nearly 50% of their trip time (on average) resting on the sea surface^5^. This compares with Scopoli’s shearwaters in the Balearic Sea that only spend 10% of their trip time resting on the sea surface. However, the criteria used here to identify the rafting behaviour of shearwaters differs from that followed by^5^ (i.e., we use a speed threshold of 0.5 m s^−1^ whilst those authors used 2.5 m s^−1^) so the percentage of trip time that individuals spend resting on the sea surface cannot be compared. Moreover, the seabird tracks reported by^5^ were recorded every minute, whilst here we have used 5-min records. As a result, the dataset used in^5^ allowed for a finer analysis. Longer rafting periods per trip provide more complete datasets for the purposes of mesoscale current pattern detection; nevertheless, the tracks analysed here were long enough to properly compare with local sea surface velocity mesoscale patterns and wind fields in order to assess the driving forces of the seabird trajectories in the Balearic Sea. The trajectories driven by slippage and Ekman drift and/or surface drag represent nearly 45% (180 trajectories) of the 405 tracks that were investigated. Thus, this method demonstrates the potential of seabird drift movements to monitor surface currents and mesoscale features in the Balearic Sea. The currents measured using seabirds can be considered to represent the combined flow of geostrophic and ageostrophic currents, the latter driven by surface Ekman and Stokes drift^5^.

The reasons for rafting behaviour by seabirds are unclear within the scientific community, despite its potential interest in terms of energy saving for individuals which could rest on the sea surface as a strategy especially if they exploit fishing discards. In any case, seabirds may rest over the sea surface simply to bathe or for plumage maintenance^33^, to digest prey after periods of feeding^34^, or just to rest and/or sleep^35^. Given that the locations of most of the observed bird drift periods are over shelf-slope regions, our results indicate that rafting behaviour of Scopoli’s shearwaters in the Balearic Sea is likely associated with their feeding. However, notice that trajectories within 5 km of the breeding colonies where seabirds tend to aggregate for non-feeding activities (such as bathing, plumage maintenance or socialising) have been rejected. Whatever the motivations for this rafting behaviour, we have shown here that GPS tracking of seabirds provides a useful approach to identify mesoscale surface currents, as well as slip and Ekman drift due to the local winds, in the Balearic Sea. However, future work should include more careful consideration of wind/wave induced effects on seabird tracks if we are to provide a more realistic estimate of the total surface current in the area. Moreover, movements of non-breeding individuals, which may spend more time resting on the sea surface, should be explored to assess whether they can provide longer drift trajectories and thus, better reproduce the mesoscale features in the region. The methodology used here can be applied over other regions and different temporal scales thanks to the large and increasing number of seabird-tracking studies worldwide^36–38^; this highlights the potential for this approach.

## Methods

### Ecology of Scopoli’s shearwaters

*Calonectris diomedea* is a medium-sized (c. 600–700 g)^39^ seabird species that breeds in colonies located on coastal cliffs and inaccessible islets across the Mediterranean basin. The birds visit their nests at night and carry out foraging trips that can last several days, reaching areas up to several hundreds of km away from the colony^40^. Foraging areas are usually located over outer continental shelf and slope waters, although birds can also forage both closer to the coast and further offshore^41^. In the Spanish Mediterranean, they breed in the Balearic Islands and also in a few islets off the Iberian coast^42^. They usually forage off the eastern Iberian and the Balearic Islands coasts, covering the entire Balearic Sea^43^.

### Bird sampling and study colonies

Fieldwork consisted of six campaigns conducted in the Balearic Sea (western Mediterranean Sea, Fig. 1) between 2007 and 2012, coinciding with the incubation (May–July) and chick-rearing periods (July–October) of Scopoli’s shearwaters (Table 1). A total of 106 breeding birds from four different colonies located in Mallorca (1), Menorca (2) and the Columbretes Islands (1) (the latter off the Valencian coast, Spain, Fig. 1) were fitted with GPS loggers to monitor their foraging movements.

Adult birds were captured at night by hand or with the help of a looped pole on the nest, when they flew back to the colony for incubation shifts or to feed their only chick. GPS loggers were attached to the back feathers using Tesa tape. Once attached, the devices weighed about 25 g, thus not exceeding the recommended threshold of 3–5% of the total bird weight^26^. Birds were recaptured within two weeks after deployment in order to remove the loggers and retrieve the data. All birds showed no sign of damage after release, and any birds that were not caught would lose the device within a few weeks due to moulting. The loggers were programmed to record a fix every 5 minutes. These protocols agree with standard guidelines of bird handling and tagging, and were approved by the competent wildlife authorities (*Servei de Protecció d’Espècies* of the Balearic Government and *Reserva Natural de les illes Columbretes* of the Valencian Government).

Some birds were either not recovered, returned without the GPS, or did not record data properly. However, for most birds (n = 75; 71%) the tracking data was safely recovered. The quantity and quality of the data collected was sufficient for an investigation of the foraging trips of tracked individuals in the study area (see tracks in right column in Fig. 2). Even though it was not the original aim of the field campaigns, we took advantage of the information gathered in this fieldwork to assess the tracks when seabirds are settled on the sea surface and act as drifting buoys driven by local winds and/or sea surface currents. These trajectories were obtained according to the sampling criteria described below. The time period analysed spans from June to September. Distances between adjacent fixes were first computed, and then the birds’ ground speed and distance to the breeding colonies (Table 1) were estimated.

### Altimeter data

We used the multi-mission (SARAL/AltiKa, Sentinel-3A, Cryosat-2, HY-2A, Jason-1, 2&3, T/P, Envisat, GFO, ERS-1&2) delayed-time (quality controlled) reprocessed Absolute Dynamic topography (ADT) gridded merged product for the Mediterranean Sea with a spatial resolution of 1/8° × 1/8°. This product is available for the time period January 1993 to the present. It is provided by the Copernicus Marine Environment Monitoring Service (CMEMS). Associated absolute geostrophic velocities derived from this product were used to compare with the trajectories of the seabirds to establish possible relations between the seabird drift data and the surface geostrophic currents.

An eddy identification and tracking tool (*py-eddy-tracker*^32^) was applied to the gridded ADT data to identify mesoscale eddies in the region of study during the seabird campaigns. The algorithm searches for closed ADT contours and then applies several tests to decide if a feature is an eddy. Version 3.0 of the eddy tracker is used. We explored the ADT contours of the identified eddies to assess the proportions of seabirds that settle inside or outside of the eddy boundaries.

### Wind product

We used the Cross-Calibrated Multi Platform version 2 (CCMPv2) gridded surface L3 ocean vector wind analysis product. This dataset^44^ is produced by combining Version-7 RSS radiometer wind speeds, QuikSCAT and ASCAT scatterometer wind vectors, moored buoy wind data, and ERA-Interim model wind fields using a Variational Analysis Method (VAM). It has a temporal resolution of 6 hours and a spatial resolution of ¼° × ¼°. Wind fields provided by this product in the western Mediterranean Sea were compared with the positions of tracked seabirds to establish a possible relationship between the drift data and the wind patterns.

### Surface buoy data

Data provided by three surface buoys from the Spanish Deep-Water Buoy Network deployed by Puertos del Estado along the eastern Spanish mainland coasts and the Balearic Islands over the 200 m isobath were utilized for validation of both surface geostrophic currents derived from altimetry and wind patterns obtained from CCMP. Namely, we used (i) the wind speed and direction at 3 m over the sea surface; and (ii) the total current speed and direction at 3 m depth as obtained from three buoys near Tarragona (40.68°N/1.47°E), Valencia (39.52°N/0.21°E) and Dragonera (39.56°N/2.10°E) for the bird sampling period (Fig. 1). Data were sampled hourly. Figure 3 shows a composite of the validation of the data corresponding to the 2012 campaign (wind data validation, upper panels and wind rose) and the three 2007 campaigns (geostrophic current validation, lower panels and current rose).

### Identification of rafting behaviour

To investigate the rafting behaviour of Scopoli’s shearwaters in the Balearic Sea we followed the behavioural movement classification provided by^26^ for this species in the region. This classification is based on both the apparent speed (m s^−1^) of GPS-tracked birds and visual inspection of trips. These authors described four categories: resting on water (<0.5 m s^−1^), feeding (0.5 to 2.8 m s^−1^), searching (2.8 to 4.2 m s^−1^) and travelling (>4.2 m s^−1^). According to this criterion, we defined rafting as two or more consecutive GPS fixes under a speed threshold of 0.5 m s^−1^, when birds are likely to be resting on the sea surface. To avoid spurious data collected when seabirds return to their colonies, an additional selection criterion was imposed: only data collected at least 5 km away from the colonies were used. Overall, we eliminated all resting trajectories shorter than 3 hours (36 consecutive GPS fixes) in order to ensure a valid comparison with both sea surface velocity patterns derived from altimetry and wind fields from CCMP. As a result, 405 trajectories were available (Table 2). An example of the application of these selection criteria is given in Fig. S1 in the Supplementary Information. This figure displays the tracked path of an individual seabird from the Columbretes colony (see Fig. 1). Its foraging trips are mainly along the eastern Spanish mainland coasts (see black paths in the map in Fig. S1). Changes larger than 150 km in the distance-to-the-colony time series (middle panel, right column) clearly indicate periods when the bird is traveling between different foraging areas. These periods are associated with velocities ranging between 5–15 m s^−1^ (upper panel, right column) and a distance between two consecutive GPS fixes (lower panel) larger than 4 km; these are clearly out of our scope. On the contrary, time periods of low variability in the distance-to-the-colony variable can be associated with smaller displacements when the bird may be feeding or resting on the sea surface. When resting the associated velocities should be lower than 0.5 m s^−1^. These are depicted by the red patches and dots in Fig. S1 and represent around 43% of total available data for this individual.

## Electronic supplementary material

Supplementary information

## Data Availability

Altimetry data analysed during this study are generated, processed and freely distributed by CMEMS (http://marine.copernicus.eu). CCMP Version-2.0 vector wind analyses are produced by Remote Sensing Systems. Data used in this study are available at www.remss.com. The *in situ* surface buoy data used to validate both the wind product and altimetry data was provided by Puertos del Estado (www.puertos.es). Version 3.0 of the *py-eddy-tracker* software used in this study is available at https://bitbucket.org/emason/py-eddy-tracker. SRTM bathymetry data were downloaded from http://topex.ucsd.edu/WWW_html/srtm30_plus.html.Data from GPS loggers attached to seabirds used to estimate the trajectories are available from the corresponding author on reasonable request.
