# Supplementary material for: Rafting behaviour of seabirds as a proxy to describe surface ocean currents in the Balearic Sea
Source: Sci Rep. 2019 Jan 10;9:17775. doi: 10.1038/s41598-018-36819-w (PMC7052213; doi:10.1038/s41598-018-36819-w)
Supplement: Supplementary file 1 — Supplementary information [file 41598_2018_36819_MOESM1_ESM.pdf]

Rafting behaviour of seabirds as a proxy to describe surface ocean currents in the Balearic Sea

A. Sánchez-Román[1,*], L. Gómez-Navarro[1,2], R. Fablet[3], D. Oro[1], E. Mason[4,1], J.M. Arcos[5], S. Ruiz[1], and A. Pascual[1]

[1] IMEDEA (CSIC-UIB), Mallorca, 07190, Spain
[2] Univ. Grenoble Alpes, CNRS, IRD, IGE, Grenoble, 38400, France
[3] labSTICC, TOMS, Brest, 29238, France
[4] Applied Physics Laboratory, University of Washington, Seattle, Washington, USA
[5] Sociedad Española de Ornitología/BirdLife, Barcelona, 08026, Spain

[*]corresponding author: asanchez@imedea.uib-csic.es

Supplementary information

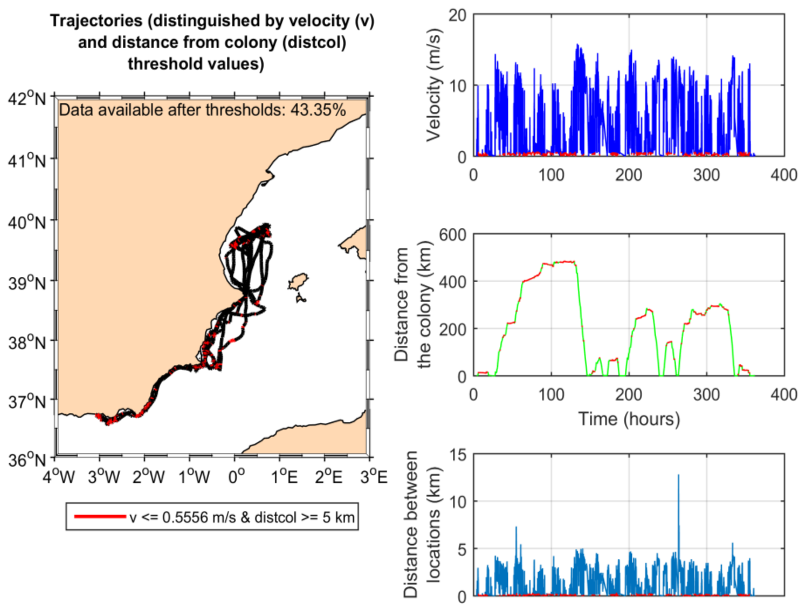

Figure S1. Example of the selection criteria applied to the trajectories (black lines) of an individual from the Columbretes breeding colony (Fig. 1). Red dots in the map stand for the tracks located more than 5 km away from the colony with tracked velocities lower than 0.5 m s^{-1} for 36 (or more) consecutive GPS fixes. Panels on the right show the ground-track velocity (m s^{-1}) derived from the GPS fixes (upper panel), the distance from the colony (km, panel in the middle) and the distance (km) between two consecutive GPS fixes (panel on the bottom). Red patches stand for GPS fixes meeting the selection criteria.
